# Early detection of memory impairment in people over 65 years old consulting at Health Examination Centers for the French health insurance: the EVATEM protocol

**DOI:** 10.1186/1471-2318-13-55

**Published:** 2013-06-06

**Authors:** Christiane Vannier-Nitenberg, Virginie Dauphinot, Bienvenu Bongue, Catherine Sass, Isabelle Rouch, Olivier Beauchet, Pierre Krolak-Salmon, Bruno Fantino

**Affiliations:** 1 Centre d’Examens de Santé de la CPAM du Rhône, Lyon, France; 2 Centre Mémoire de Ressources et de Recherche (CMRR) Lyon, Hôpital des Charpennes, Hospices civils de Lyon, Lyon, France; 3 Centre Technique d’Appui et de Formation des Centres d’Examens de Santé (CETAF), Saint-Étienne, France; 4 Département de Médecine Interne et Gérontologie clinique, CHU D’Angers, Angers, France; 5 Direction de la Performance et de la Gestion des Risques- Agence Régionale de santé de Lorraine, Nancy, France; 6 Inserm U1028; CNRS UMR5292; Lyon Neuroscience Research Center, Brain Dynamics and Cognition Team, Lyon, France; 7 University Lyon 1, Lyon, France; 8 Laboratory EA4607 SNA-EPIS, Jean Monnet University of Saint-Etienne, PRES Lyon, Saint-Etienne, France

**Keywords:** Early diagnosis, Alzheimer’s disease, Memory complaint, Mild cognitive impairment

## Abstract

**Background:**

Only half of those living with Alzheimer’s disease in France are currently diagnosed, and only one patient in three is supported during the early stages of dementia. This study aims to evaluate three cognitive tests for their predictive ability to diagnose mild cognitive impairments and Alzheimer’s disease and related disorders. For people aged 65 years or over, presenting with a memory complaint, these tests can be performed easily during a preventative consultation.

**Method/design:**

The EVATEM (*évaluation des troubles de l’équilibre et de la mémoire* (evaluation of balance and memory problems)) cohort study was designed to prospectively assess the predictive value of tests for the diagnosis of mild cognitive impairments and Alzheimer’s disease in elderly subjects aged 65 years or over. Subjects were recruited from three health examination centers that are part of the French health insurance system. If a memory complaint was identified (using a dedicated questionnaire), the five-word test, the cognitive disorders examination test and the verbal fluency test were administered during a preventative consultation. A memory consultation was performed at a University Hospital to diagnosis any potential cognitive disorder and a one-year follow-up consultation was also scheduled. We recorded 2041 cases of memory complaint at our Health Examination Centers. Cognitive tests were refused by 33.6% of people who had a memory complaint. The number of subjects sent to a University Hospital memory consultation was 832 and 74.5% of them completed this consultation. The study population therefore includes 620 subjects.

**Discussion:**

Tests for the early diagnosis of a mild cognitive impairment or Alzheimer’s disease and related disorders should be used in centers dedicated to disease prevention. These should guide subjects with memory impairment to full memory consultations at hospitals and improve the access to early medical and behavioral support.

**Trial registration:**

ClinicalTrials.gov:NCT01316562

## Background

In 2011, an estimated 5.4 million Americans were living with Alzheimer’s disease (AD); this number is expected to grow to 7.7 million by 2030. In France, AD affects about 800,000 people over the age of 75 [1]. The pathophysiological process of Alzheimer’s disease is known to begin several years before the onset of cognitive symptoms. This “pre-clinical” stage is regarded as the most efficient stage for therapeutic intervention but it is still an active domain of research [2,3]. Although the benefit of early diagnosis is still debated, early identification of a cognitive impairment could lead to earlier treatment, better care management and improved understanding of the disease [4,5]. Being able to inform the patient and their family about the disease, at a time when the patient can still fully comprehend their illness, may ensure a better long-term quality of life for patients and caregivers as well as help to limit crisis situations. Only half of those living with AD in France are currently diagnosed, and only one patient in three is supported during the early stages of dementia [6]. According to the PAQUID cohort study [7], the average Mini-Mental State Examination (MMSE) [8] score at the diagnosis of dementia is approximately 19 (maximum score possible: 30). This score corresponds to a moderate stage of the disease. The average time taken to diagnose dementia in France is estimated to be 24 months; this is relatively long as the average time taken in Europe is estimated at 20 months and, in Germany, 10 months [9]. Cognitive impairment diagnoses are performed by specialized multi-disciplinary teams during memory consultations. Diagnoses carried out in France since 2009 are listed in the Alzheimer National Bank (created under the 34^th^ measure of the National Alzheimer’s Plan (2008–2012)). Of the 118,776 patients observed in 320 memory clinics (MC) during 2010, Mild Cognitive Impairment (MCI) was diagnosed in 8.4% of the cases [10]. The health examinations centers (HEC) perform 550,000 periodic examinations dedicated to screening, prevention and health education each year; people over 60 years old represent about 20% of those receiving periodic examinations. Senior health examinations [11,12], set up in 2006, focus on screening for fall risk without particular attention to memory complaints.

A study of 1020 people aged 60 years or over, at six HEC, found a prevalence of memory complaints in 35.1% of cases [13,14]. A resulting 12.3% of the patients were sent to MC following neuropsychological tests such as the MMSE. These types of assessments are difficult to perform routinely during a preventive consultation.

Studies have therefore been set up to identify simple tests, feasible in consultation at HEC with elderly patients expressing a memory complaint, that have good predictive power to diagnose MCI [15-18] and AD and related diseases.

The EVATEM (é*valuation des Troubles de l’équilibre et de la mémoire* (evaluation of balance and memory problems)) study aims to evaluate the predictive values (PV) of three cognitive tests for the diagnosis of MCI and AD and related disorders in community-dwelling elderly people. The secondary objective is to assess the relationship between falls and cognitive impairments.

## Methods/design

### Study design

This is a prospective multicenter cohort study with a one-year follow-up. Tests were carried out at two types of medical organizations: HEC and MC. HEC are structures belonging to the French health insurance system which offers check-ups and screening for the prevention of health conditions, health education and actions, and chronic disease support. This is a free check-up and priority is given to frail or vulnerable people. MC (as defined by the circular issued by the *Direction Générale de l’Offre de Soins* (DHOS) (general directorate for healthcare provision) on 16^th^ April 2002) offer consultations and day hospitalizations which are dedicated to medical diagnostics, follow-up and nursing (treatments both with and without the administration of drugs). MC are responsible for research on AD and related disorders, participate in academic training and coordinate an interregional plan for local memory consultations.

**Figure 1:**
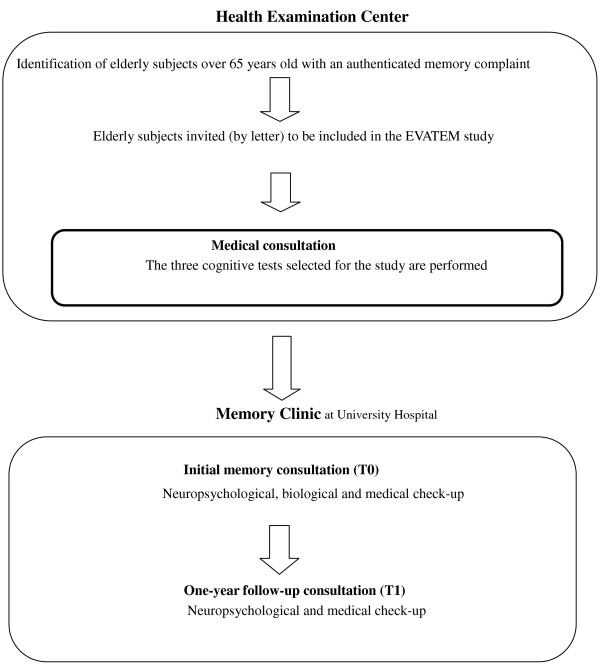
An outline of the steps in the EVATEM study.

The three HEC which participated in the EVATEM study are located in three French cities: Lyon Angers and Saint-Etienne. The diagnostics were performed through three MC in the academic hospitals of these three cities. The study is composed of several steps (Figure 1). Patients with a memory impairment were identified at HEC using questionnaires and cognitive tests during periodic health examination at T0. Memory consultations to diagnose MCI and AD and related disorders were carried out at MC according to a standardized neuropsychological evaluation and complementary exam at T0. A one-year follow-up exam, at T1, was scheduled for each of the patients.

### Ethical and legal considerations

All EVATEM subjects gave their informed consent to participate in this study. Their participation or refusal had no effect on the usual nursing performed at the HEC. The study protocol has been reviewed and approved by an ethics committee CPP Sud-Est III (*comité de protection des personnes Sud-Est III* (Persons protection comity of South-East III)) on the 7^th^ of July 2009. All procedures are in compliance with the Helsinki declaration. Data processing is approved by the CNIL (*commission nationale de l’informatique et des libertés* (National Commission for Informatics and Liberties)) under n°1362516.

EVATEM study has been registered in the clinical trials (Current Controlled Trials NCT01316562 NCT01316562).

### Population

Subjects were selected during free periodic health examinations in one of the three health examinations centers that participated in this study. These HEC were located to the cities of Lyon, Angers and Saint-Etienne.

Patients could be included if they were 65 years of age or older, lived at home or in a non-medical institution, volunteered to participate to a periodic health examination and if a memory impairment concern was identified (either by the patient themselves or by a member of their family).

Patients were excluded from the study if they had dementia, an inadequate level in French language ability, to a sensory deficiency which would compromise their evaluation.

### Sample size

We calculated the required sample size need to detect an expected sensitivity and specificity of 0.9 with the cognitive tests used in the HEC.

We set the minimum acceptable lower 95% confidence limit at 0.75, with a 0.95 probability.

Using the table of Flahault *et al.* 70 confirmed cases were required [19]. The number of controls required was calculated to be 513 with the equation: N controls = N cases [(1-P)/p] [19]; where p was the expected prevalence of MCI or AD and related disorders (we supposed this to be 12% [13])_._ The total target sample size was therefore 583.

We predicted the loss of subjects between examinations in the HEC and the MC to be 30% and so aimed to recruit 833 subjects. Actual recruitment was consistent with these estimates.

### Data collection

1. Senior health periodic examination at HEC

a. Socio-demographic data

Age, sex, marital status, address, economic affluence and vulnerability level (evaluated with the EPICES score, the individual index of deprivation used at the HEC [20]) were recorded.

a. Clinical data

We recorded weight, height, the number of different daily therapeutic groups attended, psychotropic dose (established by asking the patient or reading their last prescription), autonomy (evaluated by testing four activities of daily life [21]), and mood (evaluated using the Mini-Geriatric Depression Scale (mini-GDS) [22]).

a. Para-clinical data

Data recorded included long sighted visual acuteness (evaluated using the Monoyer scale), an evaluation of balance and walking (using tests such as the Timed Up and Go (TUG) [23] and the imagined TUG), patient proprioception sensitivity (measured using the diapason test), and the results of the handgrip test, unipodal position evaluation, chair rising, temporal analysis of walking and posture analysis (at the HEC of Lyon and Angers).

a. Memory complaint

A self-questionnaire composed of binary questions (Q1) was used to assess memory at the HEC Angers and Saint-Etienne. A questionnaire (Q2) was carried out by the doctor at the HEC of Lyon (Table 1). At least one positive answer to the questionnaire was required for the subject to be included in the study.

a. Cognitive testing performed

The EVATEM study assessed Dubois’ five-word test [24], the cognitive disorders examination test (CODEX) [25,26] and the verbal fluency test [27]. These tests were chosen as they assess verbal memory, temporal and spatial orientation and executive function but are straightforward and rapid.

Dubois’ five-word test is an episodic memory test where subjects are asked to learn five words belonging to different semantic groups and lacking phonetic similarity (for example: beverage, cooking tool, vehicular, building and insect) with control of the encoding. The test is built in such a way that it enables differentiation between symptoms of hippocampus damage (associated with AD) and common memory impairment; it includes immediate free recall exercises and postponed free recall activities. In the case of recall difficulty, a cue is suggested.

The CODEX test is designed to detect dementia in elderly subjects. It is a very simple and quick test (taking less than three minutes) and can be performed by doctors who are not specialized in the evaluation of memory or other cognitive functions. This test consists of two stages: the three-word test and simplified clock test [28] are carried out before the MMSE (five questions of spatial orientation). In the EVATEM study, the three-word test, the simplified clock test and the five spatial orientation questions were all performed.

The test of verbal fluency measures the capacity of a subject to generate a list of words belonging to the same semantic category (animals), and a list of words starting with the same letter (P), within a time limit of two minutes. This test assesses semantic memory and executive memory functions. In the case of a neurodegenerative disease, particularly in AD, semantic fluency seems to be impaired and this may be associated with possible damage to the semantic memory stock [29].

2. Neuropsychological evaluation performed at MC

The cognitive tests described below were performed routinely in MC. Episodic memory was assessed using the following tests: a free and cued recall test with 16 items (test adapted from Grober and Buschke [30]), the DMS48 [31], the Signoret battery for mnesic efficiency (BEM 144) [32] and the Birmingham Object Recognition Battery (BORB) [33]. Executive abilities were assessed using the Frontal Assessment Battery (FAB) [34], Baddeley’s dual task test [35], the Wechsler Adult Intelligence Scale III (WAIS III) [36,37], the Trail Making Test (TMT) [38], the STROOP test (GREFEX version) [39] and semantic verbal and literal fluency tests (using ‘P’ and ‘Animals’; a two minute test)[27]^.^ Instrumental efficiency was evaluated by gestural praxis assessment (symbolic gestures, pantomimes and abstract gestures), the Rey complex figure [40], the picture naming test (DO 80) [41] and the Visual Object and Space Perception Battery (VOSP) [42].

These tests were conducted by a neuropsychologist and lasted approximately 1.25 h.

**Table 1:** Memory complaint screening questionnaires (Q1 and Q2)

**Q1: self-questionnaire**	**Q2: questionnaire administered by the doctor**
	During the six last months:
1. In the last six months, have any of your close relations suggested that you have a possible memory problem?	1. Have any of your close relations told you that you have memory problems?
2. Do you sometimes suffer from a lapse of memory during daily life?	2. Have you noticed any change in your memory?
3. Do you find it difficult to calculate?	3. Do you think that your memory now works less well than memory of other people of age?
4. Do you have difficulties finding your words?	4. Do you find it difficult to orientate yourself, or did you ever not recognize a place where your close relations have told you that you have been?
5. Do you have problems remembering new information?	5. Have you ever fogotten everything about an event (even if your close relations told you about it or you saw pictures about it)?
6. Do you find it difficult to focus?	

### Diagnostic criteria for MCI and AD

The chosen definition of AD was based on the criteria of the National Institute of Neurological and Communicative Diseases and Stroke and the AD and Related Disorders Association (NINCDS-ADRDA) [43]; this can diagnose AD as possible, probable or certain. The severity of dementia was based on the MMSE score [8]. Subjects were categorized as being in the mild stage if their score was between 25 and 21, in moderate stage if their score was between 20 and 10 and in the severe stage if their score was below 10.

The diagnostic criteria for MA at a stage of dementia were the clinical criteria for probable AD [43,44].

At the stage of pre-dementia (corresponding to the amnestic MCI diagnosis [15-17,45]), diagnostic criteria were the clinical criteria of the Pre-Al study and the paraclinical criteria recently proposed for research [46,47]. For other disorders frontotemporal lobar degeneration [48], lewy body dementia [49] and vascular dementia [50] criteria were those referenced.

### Examination procedure: baseline assessment and follow-up

1. Baseline assessment: cognitive tests at HEC

The cognitive assessments were performed by trained physician and nurses. A guide, detailing how to perform the cognitive tests, specified the order in which the tests should be performed. Immediate recall and cued free test of five words was performed by the doctor, then an intercurrent test of attention for about 10 minutes and finally a free and cued delayed recall test of five words. The attention of the subject was intentionally diverted using several tests. For example, drawing pentagons as part of the MMSE and a visual-construction task in the Cognitive Assessment Battery (CAB 96) [51]. Time orientation questions (‘What day of the week is it?’; ‘What month is it?’; ‘What year is it?’), questions about drug intake (the number of therapeutic classes attended, the use of a psychotropic drug and treatment for osteoporosis), the TUG [23] and the imagined TUG were also used. The Instrumental Activities of Daily Living (IADL) scale (derived from the IADL by Lawton and Brody [21]), was used to evaluate functional capacity. This test explores the subject’s degree of autonomy or dependence in relation to four practical activities of daily life (telephone use, transportation, responsible administration of medication and budget management). The three-word test, the simplified clock test, the five questions about spatial orientation and verbal fluency tests were then performed by the nurse.

2. Baseline assessment: memory consultation at MC

An appointment for consultation at the MC was made at the end of the periodic health examination. If this had not been possible, the patient was contacted by a secretary of the HEC to schedule the appointment. The standardized biological check-up performed at HEC included a neuropsychological assessment, screening for syphilis, serum electrolytes determination and serum assays of vitamin B12, vitamin B9, albumin, pre-albumin and Thyroid Stimulating Hormone (TSH). Depression screening was performed using the Geriatric Depression Scale (GDS); a 15-item scale of depressive symptomatology suitable for elderly patients [52].

Check-ups at the University Hospital of Lyon were supplemented by the Hamilton Anxiety Scale, a questionnaire on therapeutic compliance and nutritional assessment. The established diagnosis, according to the criteria specified above, was sent to the doctor working at the HEC so that they could classify patients as “normal” subjects, subjects with MCI or subjects with dementia. A MRI scan was systematically performed at the University Hospital of Angers. At the University Hospitals of Lyon and Saint-Etienne, MRI scans were reserved for subjects where anomalies were detected during the examination.

3. Follow-up at MC

Subjects of the study will be re-called one year later (T1) at MC for a second neuropsychological check-up using the same protocol as at T0.

### Collection and data quality control

Collection and data entry are managed by the HEC of Lyon; collected data is entered by two secretaries of the HEC who have been trained in data management (double input). The collection tool was developed by the Regional Medical Service Officer (RMSO) of the Rhône-Alpes region.

### Main outcome measure

Patients will be recorded as belonging to one of three groups: “normal”, MCI or dementia subjects. The main outcome measure will be the diagnosis of MCI or AD and related disorders at T0 and T1. The secondary outcome measure will be falls at T0 and T1. A fall was defined as an event which results in a person coming to rest unintentionally on the ground, floor or at a lower level.

### Statistical analysis

Baseline characteristics of the subjects (for example, age and sex) will be described as above. Continuous variables will be summarized by the mean and standard deviation or the median and interquartile range (as appropriate). Categorical variables will be summarized as proportions of each category.

Socio-demographic and clinical comparisons will be performed between three groups (normal, MCI and dementia subjects). Group proportions will be compared using Pearson’s chi-squared test. The means of these groups will be compared using an Analysis of Variance (ANOVA) with parametric and nonparametric tests, depending on the distribution of the variables. We will study the relationship between the result of each cognitive test performed at HEC and the diagnosis confirmed by the memory consultation. A Receiver Operator Characteristic (ROC) curve will be generated for each cognitive test, using MCI and AD and related disorders diagnosis as references. The optimum threshold, providing the best compromise between sensitivity and specificity, will therefore be determined. Predictive and negative values, as well as positive and negative likelihood ratios, will be calculated. We will use a binary or multinomial logistic regression to determine which test, or combination of cognitive tests, are the most predictive for MCI and AD and related disorders diagnosis. Models will be adjusted for potential confounding variables.

A p value less than 0.05 will be considered as statistically significant.

Statistical analyses will be performed using SPSS (Statistical Package for the Social Sciences) version 17.0 for Windows (SPSS Inc., Chicago, Illinois, USA).

### Inclusions

**Figure 2:**
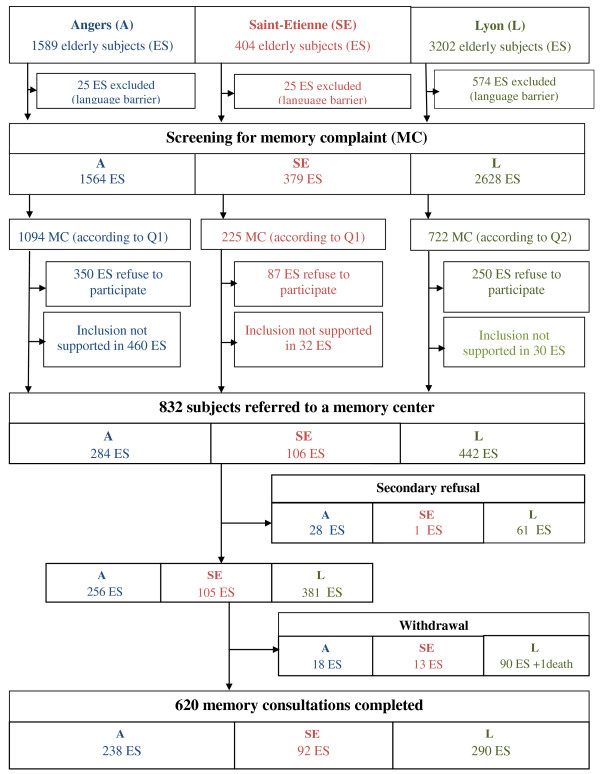
**EVATEM study flowchart.** MC: Memory Complaint, ES: elderly subjects. A: Angers, SE: Saint-Etienne, L: Lyon.

Inclusions at HEC took place from 15th November 2009 to 31st December 2011 (Figure 2). The one-year follow-up will end during the first semester of 2013.

A total of 4571 elderly subjects aged 65 years or over were eligible for research into the diagnosis of memory complaints. Amongst those subjects, 57.5% were recruited by the HEC of Lyon, 34.2% by the HEC of Angers and 8.3% by the HEC of Saint-Etienne.

The percentage of subjects with memory complaints, detected using the self-administered questionnaire (Q1), was 70% for the HEC of Angers and 59.4% for the HEC of Saint-Etienne. At the HEC of Lyon, where memory complaints were screened using a questionnaire administered by doctors (Q2), the percentage of complaints was 27.5%. Cognitive tests were refused by 33.6% of people with memory complaints. Amongst the 832 subjects for whom an appointment at MC had been made, 74.5% attended a consultation. Thus, the final study population included 620 subjects.

## Discussion

The EVATEM study will identify which one, or which combination, of these studied tests are the most predictive of MCI and AD and related disorders. This will provide a recommendation as to which should be performed on elderly patients reporting a memory complaint during a health examination at HEC.

The age for inclusion selected for the EVATEM study was 65 years old and more as the prevalence of AD is low before the age of 65 years [53]. This age criterion was also used in the PAQUID study [54] and in a three city study [55].

HEC recruit elderly patients from those listed in the files of the French health insurance system. Previous studies have compared elderly individuals using HEC to those of the general population of the same age. It appears that people over 60 years old who consult at HEC are more likely to be young male seniors (60 to 70 years old), living in couples, individuals with a healthy lifestyle (lower tobacco consumption, lower alcohol consumption and more physic activity) and those that consult more frequently than the corresponding general population (data unpublished, CETAF). However, it was previously thought that the population attending HEC was not fundamentally different from the general population of elderly persons aged over 60 years who were living at home and not suffering from disabilities.

The fact that patients agreed to participate to a periodic examination suggests that they take an interest in their health and may therefore also be more likely to attend cognitive impairment screening. Use of the Q1 questionnaire may lead to a self-selection bias associated with the fear of diagnosis, especially if another family member is suffering from AD. Representations of this type of disease and people with AD in our society are extremely negative. Consequently, patients may delay, as long as possible, addressing the issue of a disease that “crystallizes all the fears associated with aging” [6]. Societal representations lead to denial behavior and avoidance of medical consultations as the fear of diagnosis is associated with a set of losses including identity and dignity [56,57]. The administration of Q2 by doctors from Lyon led to a high selectivity in the screening of memory complaints. It will be interesting to compare the diagnostic and predictive values of the tests that result from these two groups.

This study, which began in late 2009, is based on the 1984 criteria for the clinical diagnosis of AD. In the revised NIA-Alzheimer’s Association criteria (May 2011), a semantic and conceptual distinction is made between AD pathophysiological processes and the clinically observable syndromes that result (this distinction was blurred in the 1984 criteria [58,59]). According to the revised criteria, the clinical and cognitive evaluation for MCI due to AD is almost identical to the one previously described by Petersen *et al.*[59]. All patients who would have been classified under “probable AD” by the 1984 NINCDS–ADRDA criteria would be likely to meet the current criteria for probable AD dementia. The predictive values of the three cognitive tests for the diagnosis of a mild amnesic cognitive impairment, in turn predictive of AD, will be analyzed.

The use of cognitive tests can be limited by language barriers; only people with a good level of French had been included in EVATEM. There were a particularly high number of exclusions in Lyon and it would be interested to develop a translation of the selected tests.

This study provided an opportunity for each HEC to establish care pathways with their closest MC and for medical and paramedical staff to be educated in the early diagnosis of related disorders [5]. Specific training, carried out by MC geriatricians, was required. This was well received and has demonstrated that cognitive tests can be easily integrated into a preventive consultation.

The participants of this study will be re-examined one year after their inclusion; this will enable us to estimate the predictive abilities of the tests to diagnose MCI and dementia. The use of simple tests with good predictive abilities should help guide patients from centers of prevention to MC for early medical and behavioral care management.

Further research projects are planned using the EVATEM database. These include studying the relationships between insecurity and memory complaints, cognition and anxiety-depression, cognition and walking, and cognition and vitamin D levels.

## Abbreviations

AD: Alzheimer’s disease; ANOVA: Analysis of variance; BEM 144: Battery for mnesic efficiency; BORB: Birmingham object recognition battery; CAB 96: Cognitive assessment battery; CETAF: Centre Technique d’Appui et de Formation des Centres d’Examens de Santé; CODEX: Cognitive disorders examination; DHOS: Direction Générale de l’Offre de Soins; DO 80: The picture naming test; FAB: Frontal assessment battery; HEC: Health examination centers; IAD: Instrumental activities of daily living; MC: Memory clinic; MCI: Mild cognitive impairment; Mini-GDS: Mini-geriatric depression scale; MMSE: Mini-mental state examination; MRI: Magnetic resonance imaging; PV: Predictive values; RMSO: Regional medical service officer; ROC curve: Receiver operator characteristic curve; SPSS: Statistical package for the social sciences; TSH: Thyroid stimulating hormone; TMT: The trail making test; TUG: Timed Up and Go; VOSP: The visual object and space perception battery; WAIS: Wechsler Adult Intelligence Scale

## Competing interests

The authors report no conflicts of interest.

## Authors’ contributions

BF, OB and PKS designed and managed the study. CVN carried out study follow-up and wrote the article. The follow-up of the study and revisions to the manuscript were made by VD, BB, CS, IR, PKS, and BF. All authors have read and approved the final manuscript. OB and BF obtained the funding. All authors read and approved the final manuscript.

## Pre-publication history

The pre-publication history for this paper can be accessed here:

http://www.biomedcentral.com/1471-2318/13/55/prepub
